# Evoecotoxicology: Environmental Changes and Life Features Development during the Evolutionary Process—the Record of the Past at Developmental Stages of Living Organisms

**DOI:** 10.1289/ehp.8633

**Published:** 2006-03-30

**Authors:** Jorge Herkovits

**Affiliations:** 1 Instituto de Ciencias Ambientales y Salud, Fundación Pro Salud y Medio Ambiente, Buenos Aires, Argentina; 2 Consejo Nacional de Investigaciones Científicas y Tècnicas (CONICET), Buenos Aires, Argentina

**Keywords:** biomarkers, ecotoxicology, environmental stress, evoecotoxicology, evolution, evolutionary developmental biology, ontogeny, paleoecotoxicology, paleontology, phylogeny

## Abstract

For most of evolutionary history, scientific understanding of the environment and life forms is extremely limited. In this commentary I discuss the hypothesis that ontogenetic features of living organisms can be considered biomarkers of coevolution between organisms and physicochemical agents during Earth’s history. I provide a new vision of evolution based on correlations between metabolic features and stage-dependent susceptibility of organisms to physicochemical agents with well-known environmental signatures. Thus, developmental features potentially reflect environmental changes during evolution. From this perspective, early multicellular life forms would have flourished in the anoxic Earth more than 2 billion years ago, which is at least 1.2 billion years in advance of available fossil evidence. The remarkable transition to aerobic metabolism in gastrula-stage embryos potentially reflects evolution toward tridermic organisms by 2 billion years ago. Noteworthy changes in embryonic resistance to physicochemical agents at different developmental stages that can be observed in living organisms potentially reflect the influence of environmental stress conditions during different periods of evolutionary history. Evoecotoxicology, as a multidisciplinary and transdisciplinary approach, can enhance our understanding of evolution, including the phylogenetic significance of differences in susceptibility/resistance to physicochemical agents in different organisms.

The environment has evolved from the time of Earth’s accretion at around 4.5 billion years ago (Drake and Righter 2002), permitting establishment of early forms of biota by 3.8 billion years ago. A living planet was established within these evolving environmental conditions, which resulted from abiotic inputs of chemicals, even in spite of those exerting mass extinction effects (Herkovits 2001a, 2001b). As life forms and biodiversity expanded, biologically mediated environmental processes were of increasing importance. For example, the rise of oxygen (O_2_) began about 2.4 billion years ago in the hydrosphere and the atmosphere due to the evolution of photosynthetic water-splitting capacity (Anbar and Knoll 2002). However, our understanding of the evolutionary histories linking various life forms and their environments is very limited, especially for the initial 3.3 billion years of evolution. For instance, it is assumed that prokaryotes, including those of the highest macroscopic complexity, the stromatolites, evolved for > 2 billion years. Eukaryotic cells appear to have evolved between 2.7 and 1.4 billion years ago, multicellular animals are thought to have developed about 800–700 million years ago, and the biodiversity of modern life forms, including the first known chordate, is known to have evolved by about 500 million years ago with the Cambrian explosion (Gould 1990; Meyerowitz 2002).

Sampling and preservation of the fossil record potentially bias our understanding of the evolution of biodiversity through time to an unknown degree. Moreover, recognizing fossilized biotic tissue dating to billions of years ago becomes increasingly difficult because fossils diminish in size as they increase in age. This produces such uncertainty that even a recent concept article on the origin of multicellular biodiversity suggests no date for this basic evolutionary milestone (Wolpert and Szathmary 2002). Thus, it is valuable to explore alternative possibilities that could contribute to the understanding of Earth’s evolutionary history. In this commentary I present the hypothesis that ontogenetic features of living organisms can be considered biomarkers of both the environment and living forms during the evolutionary process on Earth. Moreover, the links between metabolism, cell differentiation, and morphogenetic processes during developmental stages provide clues for a better understanding of the evolution of increasingly complex life forms through time.

Evolutionary-developmental (“evo-devo”) biology has its origin in the comparative embryology of the nineteenth century in the work of von Baer (1828) and Haeckel (1866, 1896), whose “laws” of embryonic divergence and recapitulation were put forward as being generally indicative of phylogeny. Present-day evo-devo erupted out of the discovery of the homeobox and the conservation of the spatio-temporal expression pattern of these developmental genes. It is increasingly evident from evo-devo studies ranging from plants to mammals that the evolution of distinct morphologies paradoxically relies on the reuse of a relatively small set of master regulatory genes, as exemplified in activation of the Hox genes that establish segmental patterning (Davidson 2002; Meyerowitz 2002; Swalla 2002). For example, many of the changes in plant forms have been engendered by hetero-chrony (temporal shifts in developmental pathways) or heterotopy (spatial shifts in developmental pathways).

It is noteworthy that ontogenetic trajectories are often influenced by environmental factors (the reaction norm), sometimes in abrupt ways. For example, some aquatic plants produce different kinds of leaves above and below water (heterophylly), and some insects produce winged and wingless forms at different population densities. These developmental patterns allow understanding of evolutionary divergences in metazoans and of the relationships among phyla. Here, I present the view that evoecotoxicology, the science concerned with the record in living organisms of the interactions with chemical and physical agents during the evolutionary process, provides a deeper understanding of the evolution of environment and life forms on Earth through consideration of changes in metabolic features and stage-dependent susceptibility to noxious agents during the ontogenetic process. In this commentary, the emphasis is on amphibian embryos because they are globally distributed, free-living vertebrates in direct contact with environmental conditions from the egg-cell stage onward.

## Evolutionary Environmental Change and the Metabolic Features of Embryonic Development: The Case of O_2_

It is generally accepted that the biologic and geochemical history of Earth can be separated into two supereons with regard to O_2_ (Anbar and Knoll 2002). Based on independent geochemical evidence on O_2_ availability, surface water was oxygenated between 2.4 and 2 billion years ago (Johnson et al. 1997; Rue and Bruland 1995). In the face of rising O_2_, which is usually considered the first biogenerated environmental pollutant to appear in large quantities on the planet, anaerobes died or restricted themselves to anoxic environments, whereas other organisms began using O_2_ for metabolic transformations (e.g., for efficient energy production such as mitochondrial oxidative phosphorylation). Is this critical evolutionary process registered during ontogenesis?

Amphibian embryos exhibit very marked changes in O_2_ requirements as development advances. In the egg-cell stage, O_2_ consumption is 5.7 μL/hr (per 100 embryos); at the tail-bud stage, it is 35 μL/hr; at the open mouth stage, it is 118 μL/hr; and by the complete operculum stage, it is 180 μL/hr (Herkovits and Jatimliansky 1982). Additionally, survival times in anoxia shift from > 30 hr at 2 days after fertilization to 20 hr at 10 days, to only 2–4 hr at 14 days (Adolph 1979). Taking into account that dry weights as well as total protein in amphibian embryos have similar values during all ontogenetic stages (Herkovits et al. 1983), the stage-dependent increase in O_2_ consumption appears to directly reflect metabolic changes as development advances. This conclusion is supported by a shift in mitochondrial enzymes toward aerobic metabolism at the gastrula stage (Lovtrup-Rein and Nelson 1982).

The (almost) anaerobic metabolism during early embryonic stages has been reported in a wide range of species and therefore could be a general rule in embryonic development. For instance, the energy requirements of pre-implantation embryos in mammals are generated by anaerobic metabolism (Adolph 1983; Burton et al. 2003; Robkin 1997). Also, reducing O_2_ concentration from atmospheric levels during *in vitro* culture generally improves early embryonic development across a range of species (Booth et al. 2005). In invertebrates, the same pattern of low O_2_ uptake by early-stage embryos occurs. For example, intertidal crabs exhibit a greater than 10-fold increase in O_2_ consumption by the time of hatching (Taylor and Leelapiyanart 1997). Artemia could be a paradigmatic example in that 60% of early-life-stage embryos can survive for 4 years of continuous anoxia at physiologic temperatures (Clegg 1997).

These examples of anaerobic metabolism at early developmental stages in different species are usually interpreted as serving to protect the embryo from oxidative stress and/or as an array of adaptations that enable them to survive a wide variety of environmental extremes. However, in present-day aerobic conditions, there is no apparent adaptive advantage justifying the highly conserved occurrence of anaerobic metabolism restricted to initial developmental stages. In this context, anaerobic metabolism in early development could be interpreted as providing support for the evoecotoxicologic hypothesis that living organisms recapitulate metabolic features of their evolutionary history at ontogenetic stages.

These features can also be considered as biomarkers of evolution of the environment exemplified by the transition from anoxic to oxic conditions on Earth. Thus, from an evo-ecotoxicologic perspective, the evolution to multicellularity has its origin in the anoxic world > 2 billion years ago. In fact, the blastula-stage embryo could reflect the existence of multicellular organisms in the archaean anoxic world. Moreover, taking into account that intercellular adhesiveness exists in protists and occurs in amphibian embryos early in the blastula stage (Herkovits 1978), the earliest multicellular life forms appear to be an ancient achievement in the anoxic Earth. Anaerobic multicellular organisms exist today, but their complexity is limited compared with that of aerobic metazoans. In any case, it is noteworthy that the recapitulation of ancient-life features, such as anaerobic metabolism during early embryogenesis, seems to be essential for the accomplishment of normal ontogenesis even for vertebrates.

From an evoecotoxicologic perspective, the transition to aerobic metabolism at the gastrula stage in extant organisms corresponds to a period of major progress during the history of multicellular life in the rising free O_2_ environment. In the case of amphibian phylogeny, this may be reflected by the rearrangement of cellular organization toward tridermic organisms and within this process the evolution of early chordate life forms. Regarding the timing of adaptation to free O_2_ during the evolutionary process, the gradual increase in O_2_ consumption as embryonic development advances (Herkovits and Jatimliansky 1982) could reflect *a*) a gradual change in the amphibian ancestor’s habitat, *b*) movement toward more oxic environments as metabolic adaptation allowed, or *c*) an increasing capacity to use a resource already existent at a high level. Based on a battery of studies including phylo-genetics, structural biology, protein engineering, metabolism, competition, and genomics, it has been concluded that the ability to adapt to environmental conditions was already in place 3.5 billion years ago (Zhu et al. 2005). Therefore, the first option or a combination of the first two seems more likely and probably occurred as a multistep process.

Thus, an evoecotoxicologic interpretation places the development of aerobic metabolism-dependent evolutionary achievements, including aerobic multicellular organisms, at least 1.2 billion years in advance of their appearance in the available fossil records. Based on this construction, the creatures from the Ediacaran, Tommotian, Oman, Chengjiang, and Burgess Shale can be viewed as part of a broader picture with roots in multicellular life forms in deep anoxic Earth history. The change, then, from anaerobic to aerobic metabolism in early embryogenesis seen in both vertebrates and invertebrates today serves as an evoecotoxicologic biomarker of the transition from anoxic to oxic Earth.

The colonization of terrestrial habitat by amphibians about 380 million years ago (Carroll 1987) required a change from uptake of O_2_ in water to uptake from air. This change reduced the energy requirement for O_2_ uptake because of the increased concentration of O_2_ in air and the increased mobility of the medium over respiratory surfaces. A possible biomarker of access to this higher O_2_ environment and the concomitant need for enhanced protection against increasing oxidative stress is the novel and more efficient glutathione *S*-transferase isoenzyme, which is highly expressed in the postmetamorphosis amphibian liver (Amicarelli et al. 2004). It is well accepted that this last evolutionary step is related to O_2_ consumption and, in amphibian phylogeny, corresponds to a process of adaptation to occupy a new niche, the terrestrial habitat.

The access to higher free O_2_ levels seems to have produced, probably at least 200 million years ago, living organisms with homeothermic metabolism (Carroll 1987). It is noteworthy that both mammalian and avian embryos have poikilothermic metabolism until birth (homeothermic condition is provided by parent organism). The usual explanation of this fact is based on the energy needs of the poikilothermic embryo, which are considerably less than in comparably sized homeothermic organisms. Although for present environmental conditions this explanation could be acceptable, from the evoecotoxicologic perspective, the poikilothermic metabolism of avian and mammalian embryos may reflect the evolutionary history of multicellular life from Earth’s anoxic period through to the terrestrial invasion and the eventual development of homeothermic metabolism.

Because the shift from anaerobic to aerobic metabolism in evolution as well as ontogeny is directly related to mitochondrial functions, it seems appropriate to comment on mitochondria in eukaryotic organisms from the evoecotoxicologic perspective. According to mainstream scientific literature, the acquisition of mitochondria in eukaryotic life forms is estimated to have occurred about 2 billion years ago (Meyerowitz 2002). However, mitochondrial activation of aerobic metabolism at the gastrula stage seems to support the assumption that these organelles existed in eukaryotic cells long in advance of the achievement of oxidative phosphorylation. As a corollary of this hypothesis, the adaptation of mitochondria toward aerobic metabolism probably occurred in eukaryotic organisms. It is noteworthy that, based on phylogenetic studies, mitochondria are believed to have originated about 3.5 billion years ago (Zhu et al. 2005) in the early anoxic Earth. Mitochondria and hydrogenosomes are believed to be aerobic and anaerobic homologues of the same endosymbiotically derived organelle (Hrdy et al. 2004). Moreover, mitochondria are present in living anaerobic life forms, supporting the evoecotoxicologic interpretation that mitochondria probably evolved from anaerobic to aerobic metabolism within eukaryotic organisms.

## Are Environmental Changes during Evolutionary History Reflected in Stage-Dependent Susceptibility to Noxious Agents?

It is generally accepted that changes in environmental conditions at global and regional levels during Earth’s evolutionary history were driving forces for the evolution of living forms and that resulting ecosystem features lead to the establishment of a living planet (the Gaia hypothesis) (Lovelock 1979). However, our understanding of the adaptation of living forms to specific environmental features during evolutionary history is far from complete. For instance, although there are no documented mass extinction events before the late Precambrian catastrophe, it is generally accepted that because of aggressive environmental conditions, life probably had to evolve several times before it was definitively established on the planet. Taking into account that at least five outstanding mass extinction events were registered during the past 600 million years (Raup and Sepkoski 1984), it seems logical to assume that a higher number of yet undocumented mass extinction events occurred during the pre-Phanerozoic Eon, which had a duration about six times longer. For those ancient times, interpretation of environmental scenarios can differ markedly, even for the well-documented rise of free O_2_ about 2.4 billion years ago, which probably resulted in the extinction of a significant part of the anaerobic biodiversity. For instance, the classical argument that deep oceans became oxidized 1,800 million years ago is based principally on the disappearance of banded iron formations in the stratigraphic record (Johnson et al. 1997; Rue and Bruland 1995). An alternative explanation could be that the deep oceans became sulfidic, rather than oxic, and under these conditions biologically important trace metals would have been scarce in most marine environments, restricting primary productivity and limiting the distribution of eukaryotic algae (Anbar and Knoll 2002). In any case, there is no doubt that the early living forms had to cope with a wide range of environmental stresses. Some stresses, such as the lethal ultraviolet B (UV-B) irradiation before the ozone shelter was established, persisted for as long as 2 billion years.

It is generally believed that after each environmental crisis, including mass extinction events due to chemical explosion scenarios, a relatively pristine environment developed, allowing the surviving species to radiate (Herkovits 2001a, 2001b). From the evoecotoxicologic perspective, these major up and down episodes in environmental stress conditions should be reflected in the ontogeny of living organisms. Could the well-documented stage-dependent susceptibility to physicochemical agents during developmental stages (Degitz et al. 2000; Fort et al. 2004; Greulich and Pflugmacher 2003; Herkovits and Fernandez 1979; Herkovits et al. 1997; Kast-Hutcheson et al. 2001; Rutledge 1997) provide a second major link between ontogenesis and the adaptation of life forms to their environment? The high resistance at the blastula stage to physicochemical stress (Herkovits et al. 1997; Pérez-Coll and Herkovits 1996), enhanced in free-living embryos by protective barriers such as the vitelline membrane and jelly coats, provides support for the argument that this stage reflects very aggressive environmental conditions during the evolution of early multicellular organisms. Thus, the absence of ozone protection against UV-B (developed only after free O_2_ was available, probably starting about 2.4 billion years ago) may be reflected by the very high resistance of amphibian embryos at early developmental stages to UV-B (Herkovits J, unpublished data) as well as to other agents exerting oxidative stress (Herkovits et al. 1997; Pérez-Coll and Herkovits 1990; Vismara et al. 2001). This resistance, characteristic of amphibian early embryo stages, is in line with the evoecotoxicologic hypothesis that ontogenetic stages in living organisms can be considered biomarkers of major features of both the environment and life forms during the evolutionary history and provides additional support to the suggestion that metazoan organisms existed in the ancient anoxic Earth.

In evaluating these early developmental stages (e.g., the blastula) for relative resistance to aggressive environmental conditions, there is also a stage-dependent susceptibility (Bustuoabad et al. 1977) that potentially reflects changing environmental conditions during the evolution of multicellular life forms in the anoxic Earth. This fact is not surprising taking into account that, from an evoecotoxicologic perspective, blastula-like organisms could have existed during hundreds if not more than a thousand million years of evolution in the anoxic Earth. As a whole, high resistance to environmental agents during initial developmental stages contrasts with lowered resistance as cell differentiation and morphogenetic processes achieve increasing complexity (Bogi et al. 2003; Christensen et al. 2005; Herkovits et al. 1997; Pérez-Coll and Herkovits 1990, 1996; Vismara et al. 2001). Some agents or combinations of agents with novel mechanisms for adverse effects probably represent exceptions to this generalization. However, the fact that early organogenic-stage embryos in general are very susceptible to noxious agents [in spite of high capacity to recover from adverse effects (Herkovits 1977; Herkovits and Faber 1978; Herkovits and Fernandez 1979)] contributes to the vision that the increasing complexity of cell differentiation and morphogenesis is associated with relatively low environmental stress conditions during evolution that are reflected in ontogeny. It is noteworthy that metamorphosis, also a complex cell differentiation and morphogenetic process in both invertebrates and vertebrates, is another period of very high susceptibility to a variety of environmental agents (Howe et al. 2004; Wilson 2004; Zhang 2002).

From a global perspective, probably the worst environmental stress conditions occurred during early periods of Earth’s history and during mass extinction events. However, even during the “bonanza” times, because of limited abiotic inputs (e.g., volcanism) and the increasing biochemical warfare among species as bio-complexity advanced, for example, herbivory (Pérez-Coll and Herkovits 2004), the adaptation to physicochemical agents continued. An example of such a gradual shift in resistance to physicochemical stress can be observed in amphibian larvae within the first weeks after complete operculum stage as resistance to metals increases (Herkovits J, unpublished data). Because such changes in susceptibility at different developmental stages have no apparent adaptive advantage for present environmental conditions, they are in line with the evoecotoxicologic hypothesis that stage-dependent resistance to physicochemical agents reflects the evolutionary history of living forms in an evolving environment. On the other hand, resistance to environmental agents can change significantly even within each developmental stage, as has also been reported for the amphibian blastula (Bustuoabad et al. 1977). The changes in ontogenetic susceptibility to physicochemical agents and resulting key features of metabolism as biomarkers of evolution may justify a reevaluation of the criteria by which developmental stages are defined.

It is generally accepted that environmental stress can select resistant individuals within species and resistant species within ecosystems. Within this context, resistance to physicochemical agents potentially exhibits particular features in different species according to their exposure histories and response capacities acquired during evolution. Resistance, therefore, should be considered on a case-by-case basis. The organism’s strategies to resist adverse effects of physicochemical agents could be related to toxicologic characteristics of those agents. For instance, the introduction of new insecticides has resulted in a rapid increase in resistance among many species to multiple classes of insecticides. The remarkable homology of the genes associated with this, as shown by intron sequencing and by the presence of a conserved transposable element, indicates that the resistance allele (e.g., for cytochrome P450 in the case of DDT) likely had a single origin and subsequently spread around the world (Dabron et al. 2002; Denholm et al. 2002). Even low-level exposures could enhance resistance against adverse effects, including lethality (Calabrese and Baldwin 2003; Herkovits and Pérez-Coll 1995). On the other hand, in heavily contaminated places, significant adverse effects on biota occur in a few years (Herkovits et al. 1996). These examples of environmental stress conditions shaping changes in biota could be expanded by using the evoecotoxicologic approach to study the evolution of both living forms and environmental features on Earth. This potentially provides for a better understanding of the profound impact of environmental features on living organisms, as well as the role of living organisms in shaping the environment. Some biomarkers of environmental conditions, such as those related to the rise of O_2_ starting about 2.4 billion years ago and subsequent changes toward an aerobic metabolism, have global significance and therefore might be expected to be evident in all aerobic organisms at specific developmental stage(s) according to their phylogenetic trajectory. Conversely, those related to very local features, such as the case of serpentinite-hosted hydrothermal field beneath mid-ocean ridge (Kelley et al. 2005), would be expected to be evident only in organisms living in those particular environmental conditions.

Environmental toxicology and chemistry have been extended to the interpretation of the fossil record using a case study focusing in the Cretaceous–Tertiary mass extinction event (Herkovits 2001a, 2001b). Here, evoecotoxicology is introduced as a multidisciplinary and transdisciplinary approach that can contribute to a better understanding of evolutionary history, as well as to the susceptibility/resistance features of living organisms to physicochemical agents.
